# β-glucan combined with PD-1/PD-L1 checkpoint blockade suppresses pancreatic tumor growth after ablation therapy

**DOI:** 10.1016/j.clinsp.2025.100805

**Published:** 2025-10-12

**Authors:** Shengbo Wu, Xixi Sun, Han Wang, Jiayuan Chai, Bin Huang

**Affiliations:** Department of Ultrasound, Zhejiang Hospital, China

**Keywords:** Pancreatic cancer, Beta-glucan, Programmed cell death, Dendritic Cell, Ablation Technique

## Abstract

**Background:**

To explore whether β-glucan combined with anti-PD-L1 reverses the immunosuppressive Tumor Microenvironment (TME) in residual pancreatic cancer after Microwave Ablation (MWA).

**Methods:**

The effect of β-glucan on the activation of Dendritic Cells (DCs) was assessed in vitro. Subcutaneous and orthotopic pancreatic tumor models with incomplete MWA were treated with β-glucan, anti-PD-L1, or both. Tumor growth, immune cell populations in TME, and survival were analyzed.

**Results:**

β-glucan elevated MHCII+DCs, meanwhile combination therapy increased the CD8+/Treg ratio, suggesting enhanced anti-tumor immunity. This immunomodulatory effect translated into the combination therapy demonstrated significantly greater efficacy in suppressing residual tumor growth and prolonging overall survival.

**Conclusion:**

β-glucan administration increased MHCII+DCs, and in combination therapy, this led to an elevated CD8+/Treg ratio, enhancing the anti-tumor immunity. β-glucan therapy reversed the immunosuppressive TME in pancreatic cancer following MWA ablation, thereby enhancing the anti-PD-L1-associated antitumor responses. This resulted in a significant suppression of residual tumor growth, leading to prolonged survival in mice establishing orthotopic pancreatic tumors.

## Highlights

• First proposal of “DC-trained immunity combined MWA therapy” for pancreatic cancer.
• Trained DCs reshape post-ablation immunosuppressive tumor microenvironment.
• High clinical potential with broad applicability to improve ablation outcomes.

## Introduction

Pancreatic carcinoma is likely to be the second leading cause of cancer-related death, causing a 5-year survival rate of less than 7 %.^1^ For more than 70 % of patients diagnosed with advanced pancreatic cancer, radical treatments such as surgery are not available.^2^ Therefore, minimally invasive interventional therapy has become a viable option for unresectable pancreatic cancer. More importantly, MWA is a reliable therapeutic method for pancreatic cancer, which has been approved in clinical applications.^3^ However, high recurrence rates and incomplete ablation are the urgent problem for microwave ablation. Cumulative evidence has proved that residual tumors after MWA have an immunosuppressive TME, leading to rapid deterioration.^4^^,^^5^

Currently, the intersection between energy-related ablation and cancer immunotherapy is broadening rapidly.^6^ Immune Checkpoint Blockade (ICB) therapy has been increasingly studied in recent years and showed an exciting new approach to cancer immunotherapy. Unfortunately, research has shown that pancreatic cancer after ablation is prone to becoming resistant to ICB therapy, which is caused by the tumor's immunosuppressive microenvironment.^7^ Thus, immune reactivation in the TME is key to improving the antitumor effect in combination therapy of MWA and ICB.

Immune agonists hold tremendous potential for tumor treatment via activating TME.^8^ As an immune adjuvant, β-glucan extensively modulates the immune system by training myeloid cells, particularly macrophages, and enhancing antitumor NK-cell responses in cancer.^9^^,^^10^ Previous studies have demonstrated that yeast-derived granules β-glucan can be aggregated and transported to the pancreas by intraperitoneal injection, showing features of training immunity to intrapancreatic myeloid cells.^11^ Moreover, its immunity training effects on DCs were well-established in the literatures.^12^^,^^13^ The process of innate immune cells like DCs after epigenetic rewiring, transcriptomic and metabolic to display memory-like characteristics termed “trained immunity”.^14^ The increased levels of epigenetic marks in trained cells include H3K27ac, H3K4me3 and H3K4me1, which promoted inflammatory responses and immunological signaling^15^ by stimulating the open promoter regions and enhancer sites of genes.^16^ In particularly, trained immunity favor of the production and release of proinflammatory cytokines, such as IL-6, IL-1β and TNF-α, by innate immune cells.^17^ Enhanced mature Dendritic Cells (DCs) enhance costimulatory molecule expression and produce cytokines and chemokines essential for effective T-cell activation, thereby inducing antitumor immunity and facilitating migration into lymphoid tissues and tumor.

To address the disadvantages of ICB therapy discussed above, we sought to investigate the use of β-glucan plus anti-PD-L1 as antitumor therapy. DCs, as the key cellular link between innate and adaptive immunity, play a significant role in enhancing adaptive T-cell responses.^18^^,^^19^ Previous research has shown that β-glucan could train DCs and mediate a long-lasting effect that enhances T-cell responses for up to 1 year.^20^ We hypothesized that β-glucan would enhance the concentration of DCs in the TME, which would reverse the tolerance of anti-PD-L1 therapy. To this end, we presented a pancreatic cancer experimental model to determine the therapeutic efficacy of β-glucan combined with anti-PD-L1 in mice after MWA. The purpose of the study was to determine whether β-glucan combined with anti-PD-L1 could be used to reinvigorate and augment ICB therapy in patients with cancer after MWA.

## Materials and methods

### Materials

All chemicals in our study were purchased from Sigma-Aldrich unless other instructions. β-glucan was provided by Apeptide (Shanghai, China). Anti-PD-L1 was purchased from Bioxcell (B7-H1, Catalog No. BE0101).

### Mice and cell line

The Pan02 cell line was originally obtained from the American Type Culture Collection (ATCC) and used in the experiment were cultured in the recommended medium and condition. Female and male C57BL/6 mice aged six to eight weeks were utilized in all experiments, which were procured from Shanghai Yuanchuang Biotechnology Co., Ltd. Mice were maintained on a 12 h dark/light cycle with controlled humidity (around 55 %). All procedures were approved by the Institutional Animal Care and Use Committee of the Laboratory Animal Center of Shanghai Tenth People’s Hospital and complied with ARRIVE guidelines.

### Bone marrow-derived DC (BMDC) maturation

Bone marrow‑derived DC (BMDC) were obtained from the femurs and tibias of C57BL/6 mice as described previously.^21^^,^^22^ Different concentrations of β-glucan were added to the cultures. After incubating for 24 h, 10 µL of CCK-8 solution was added to detect cell viability.

The activation and maturation of BMDCs driven by different formulations were analyzed by detecting the expression of cell-surface markers on BMDCs. BMDCs were incubated with β-glucan (10, 50 and 100 μg) for 24 h. The surface marker expression of collected BMDCs was evaluated with flow cytometry using PE-conjugated anti-CD11c, FITC-conjugated anti-CD80, APC-conjugated anti-CD86, and FITC-conjugated anti-IA/IE (MHC II) antibodies according to previously described method.^22^ Data were collected by FCM (BD, Fortessa X20) and analyzed using FlowJo Software.

### Cytokine detection

The concentration of bioactive TNF-α, IL-6, IL-1β and IFN-γ in serum and cell culture supernatant was tested by Enzyme-Linked Immunosorbent Assay (ELISA) kit. All the test kits were obtained from MultiSciences.

### Western blot procedures

To verify the BMDM were trained by β-glucan. All protein samples (using the Enhanced BCA Protein Assay Kit, Beyotime, P0010) obtained by cells were mixed in SDS-PAGE loading buffer (Beyotime, P0015L) with heating at 100 °C for 10 min. After gel electrophoresis and protein transfer, the Polyvinylidene Fluoride (PVDF) membrane was blocked with 5 % nonfat milk, and then the protein was incubated overnight with 1:1000 diluted Acetyl-Histone H3 (Lys27) rabbit monoclonal antibody (Beyotime, GTX128944), Histone H3 mouse monoclonal antibody (Beyotime, AF0009) and GAPDH mouse monoclonal antibody (Beyotime, AF0006) at 4 °C. After washing with tris-buffered saline containing 0.5 % Tween 20 (Solarbio, China), HRP-conjugated goat anti-rabbit IgG (Beyotime, A0208) or goat anti-mouse (Beyotime, A0216) IgG was added. The membranes were then incubated for 1 h at room temperature. Finally, an automatic chemiluminescence image analysis system (Tanon 4600) was performed to detect immunoreactive proteins.

### In vivo tumor models and treatment

Pan02 cells (1 × 10^6^) suspended in 100 μL phosphate-buffered saline were subcutaneously injected into the left flanks of C57BL/6 mice (6 − 8 weeks old). When the volume of the tumor grew to 400 mm^3^ (day 21 after implantation), an incomplete ablated mouse model was established using a microwave therapy device (ECO-100E, Yigao Microwave Electric Institute, Nanjing, China) (5 W, 1‒1.5 min). And then mice were randomly assigned to 4 experimental groups: control (PBS), β-glucan (1 mg/mice), anti-PD-L1 (3.75 mg kg^-1^), or β-glucan + anti-PD-L1. The tumors were carefully measured with a digital caliper every other day, and the volume (mm^3^) was calculated as the formula: long diameter × short diameter2)/2. Mice with established subcutaneous Pan02 tumors were euthanized when the tumor volume reached 1000 mm^3^.

For the orthotopic Pan02 model (PBS group, β-glucan + anti-PD-L1 group), treatment was initiated when the subcutaneous tumor volume increased to approximately 200 mm^3^. Then, the residual tumor burden and the progress of the tumor were monitored by bioluminescent signals.

### Flow cytometry

The tumor tissues were treated with the tissue dissociation kit (Miltenyi Biotec, Germany) to digest into a single-cell suspension. The harvested cells were resuspended in a staining buffer, blocked with the Fc-blocking monoclonal antibody, and then further stained with several fluorochrome-conjugated antibodies against CD45-eF506, CD11c-PE-cy7, MHCII-FITC, CD86-APC, CD206-PE, CD3-PE-Cy7, CD4-FITC, CD8-PerCP-Cy5.5, CD25-APC, Foxp3-PE and then detected by FCM (BD, Fortessa X20).

### Immunohistochemistry

CD8, MHCII, and FoxP3 were labeled using IHC. Tumors harvested at the end of the experiment were fixed in 10 % buffered formalin and then tissues were subsequently embedded in paraffin. After baking for 90 min at 68 °C deparaffinizing, antigen retrieval was experimented with 10 × 10^–3^ M sodium citrate (pH = 6.0, at 100 °C for 10 min). Then quenching the endogenous peroxidase activity and blocking the section, incubating overnight with primary antibodies at 4 °C, further incubating with a biotin-conjugated secondary antibody for 1 h at room temperature. Finally, representative images of each group of tumors were captured through a fluorescent scanning camera (KFBIO, KF-TB-400).

## Statistical analysis

The quantitative results were expressed as mean ± SD. All statistical differences were performed by GraphPad Prism software to use variance (ANOVA) for comparing two or multiple groups, Student’s *t*-test, or Tukey’s posttest for normal test. The survival curve was performed by the Log-rank test. The statistical significance was set as ns, not significant, * *p* < 0.05, ** *p* < 0.01, *** *p* < 0.001.

## Results

### β-glucan stimulated trained immunity and promoted the maturation of BMDC

**Fig. 1:**
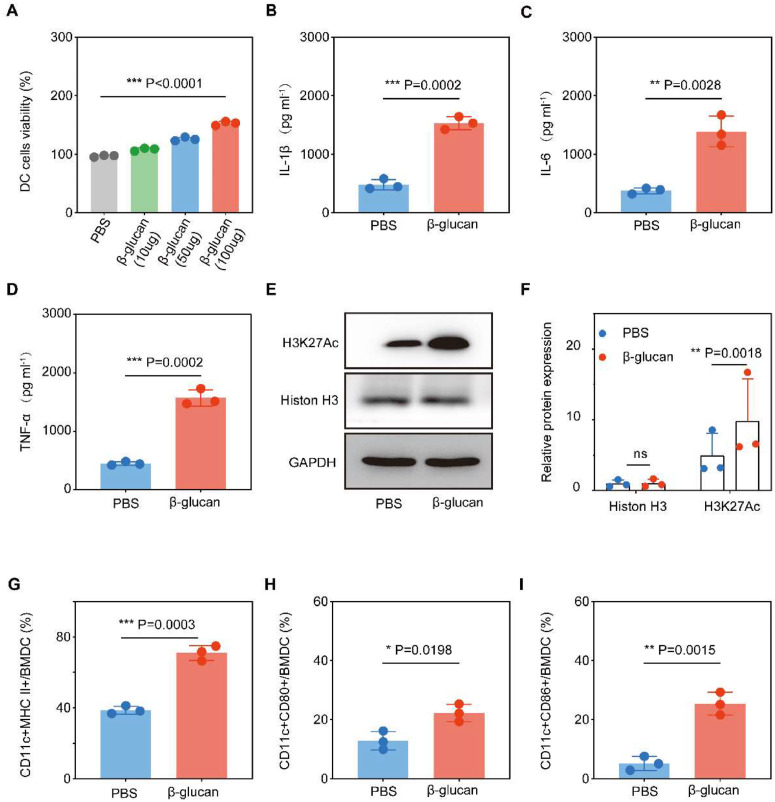
**β-glucan stimulate BMDC trained immunity and maturity.** (a) Viability of BMDCs after incubation with different dose of β-glucan for 24 h. (B‒D) TNF-α, IL-6 and IL-1β production by BMDCs after β-glucan -trained (*n* = 3) or PBS (*n* = 3) stimulation assessed by ELISA. (E‒F) H3K27Ac and Histon H3 expression in PBS (*n* = 3) or β-glucan-stimulated (*n* = 3) DCs. Representative western blot and summarized data. (G‒I) Flow cytometry analysis of the ratio of CD11c+MHCII+/BMDC, CD11c+CD80+/BMDC and CD11c+CD806+/BMDC. The cells were co-incubated with 100 µg β-glucan for 24 h.

To determine the effects of β-glucan on DCs, we performed a training experiment in vitro. First, whether β-glucan was toxic to BMDCs was evaluated with a cell viability assay. The results showed that the different concentrations of the β-glucan did not show any cytotoxicity, but rather showed a dose-dependent promotive effect on the DCs (Fig. 1A). Respectively, the extent of DC maturation and the related cytokine secretion were evaluated by FCM and ELISA. Cytokines TNF-α, IL-6, and IL-1β were surrogate markers to measure the trained immune response.^23^ β-glucan-trained DCs showed enhanced pro-inflammatory cytokine production compared to untreated DCs (Fig. 1B‒D), showing that β-glucan induces trained immunity. β-glucan training was also related to metabolic reprogramming as indicated by significantly increased H3K27Ac expression (Fig. 1E‒F), consistent with previous findings.^24^

To further assess the effects of β-glucan on BMDC maturation, the expression levels of CD80, CD86, and MHC II on the cell surface were analyzed by flow cytometry after incubation for 24 h. Compared with PBS treatment, stimulation with β-glucan significantly promoted the expression of surface markers on mature BMDCs (Fig. 1G‒I). These results suggested that β-glucan could effectively activate BMDCs.

### β-glucan combined with anti-PD-L1 against post-surgery tumor progression

**Fig. 2:**
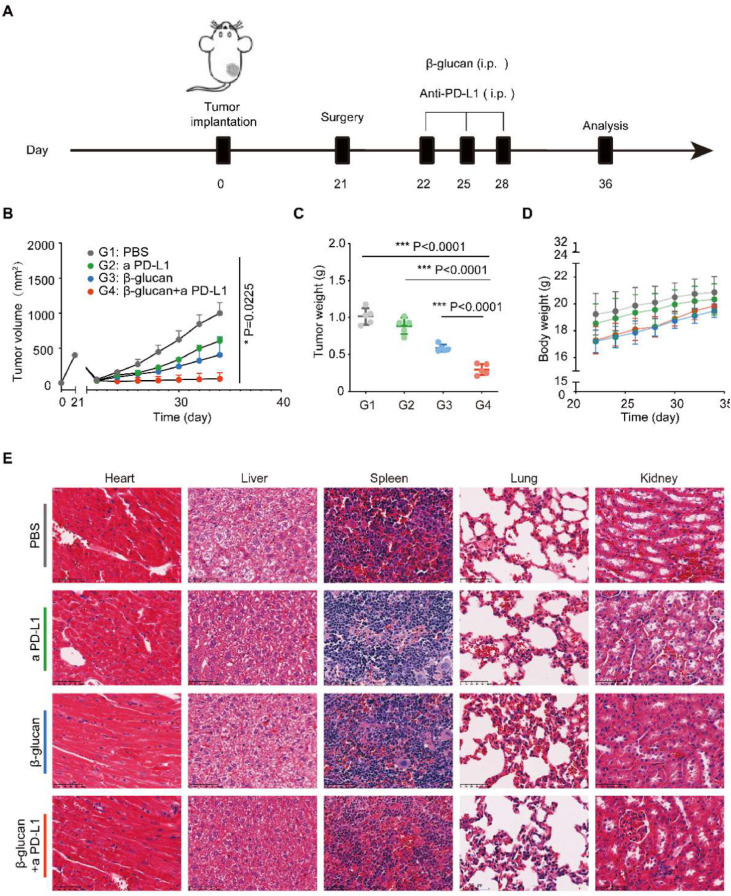
**β-glucan combined with anti-PD-L1 against post-surgery tumor progression.** (A) Schema for in vivo β-glucan therapy in a tumor mouse model receiving incomplete MWA therapy. (B‒C) Average tumor growth curves and weights of tumor after various treatments (*n* = 5). (D) Time-dependent body weight surveillance of mice after different treatments (*n* = 5). (E) Representative H&E stained tissue sections of three biologically independent animals from each group (Bar = 100 μm). Statistical difference was calculated using two-tailed unpaired Student’s *t*-test. Data were expressed as means ± *S*. * *p* < 0.05, ** *p* < 0.01 and *** *p* < 0.001.

To investigate the antitumor effects of β-glucan combined with anti-PD-L1 in C57BL/6 mice bearing pancreatic cancer after surgery, we separated the mice into 4 treatment groups (including Control group [PBS], anti-PD-L1 group, β-glucan group, and β-glucan + anti-PD-L1 group). Considering the tendency of dextran to the pancreas,^11^ the β-glucan and β-glucan + anti-PD-L1 groups received a dose of β-glucan = 1 mg/mice once every 3-days via intraperitoneal injection 1-days after surgery for 3 times (Fig. 2A). We found that combination therapy had a significant tumor growth inhibition effect when compared to the controls and single therapy (Fig. 2B‒C). Moreover, no abnormal weight reduction of mouse was suggested in the β-glucan plus anti-PD-L1 treatment group (Fig. 2D). To assess the biosafety of this combination immunotherapy, pathological analysis of major organs was also tested (Fig. 2E).

**Fig. 3:**
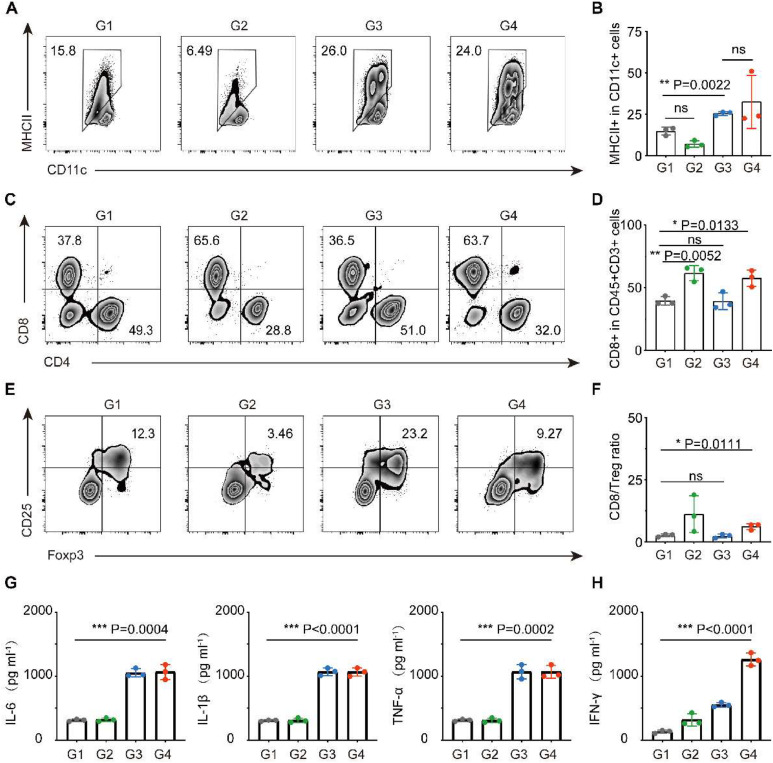
**Study on anti-tumor mechanism of β-glucan in vivo.** (a‒f) Representative flow cytometric analysis and relative quantification of DCs (CD11c+MHCII+) (A‒B), CTL (CD8+CD3+CD45+) (C‒D), and Treg (Foxp3+CD25+CD4+) (E‒F). (G‒H) Cytokine levels of TNF-α, IL-6, IL-1β (G), and IFN-γ (H) in the serum after various treatments (*n* = 5). Group 1: PBS, Group 2: a PD-L1, Group 3: β-glucan, and Group 4: β-glucan + *a* PD-L1. Statistical difference was calculated using two-tailed unpaired student’s *t*-test. Data were expressed as means ± *S*. * *p* < 0.05, ** *p* < 0.01 and *** *p* < 0.001.

We then examined the immune cells in the TME and found that in both combination therapy and β-glucan single group, the infiltration of DCs (CD11c+MHII+) was significantly increased (Fig. 3A‒B). Benefit from trained DCs, the FCM showed that combination therapy could significantly increase the infiltration of cytotoxic T-cells (CD45+CD3+CD8+) in tumors (Fig. 3C‒D). Concomitantly, the indicator of anticancer immune balance (CD8/Treg) was elevated to a higher ratio in mice receiving combination therapy (Fig. 3E‒F). In addition, cytokines in serum including TNF-α, IL-6 and IL-1β that play pro-inflammatory activation and IFN-γ play anti-tumor roles increased cellular immunity significantly, which again performed a strong immune response induced by β-glucan treatment (Fig. 3G‒H).

### Combination therapy inhibits the growth of pancreatic cancer in orthotopic models

**Fig. 4:**
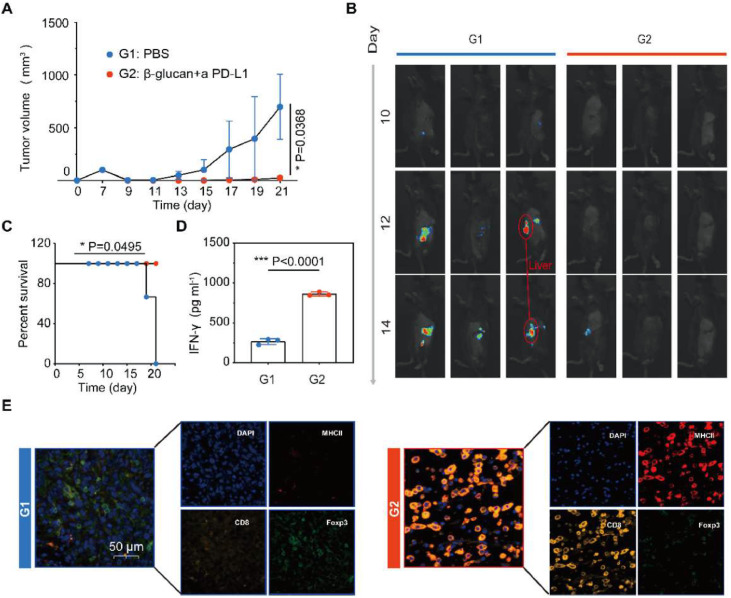
Combination therapy inhibits the growth of pancreatic cancer in orthotopic models. (A) Average tumor growth curves of tumor after various treatments (*n* = 3). (B) Representative bioluminescence images of Luc^+^Pan02 in the orthotopic tumor after various treatments as indicated. (C) Kaplan-Meier survival curves of Pan02 tumor-bearing mice after varied therapeutic combinations (*n* = 3). (D) Cytokine levels of IFN-γ in the serum after various treatments (*n* = 3). (E) Polychromatic immunofluorescent staining images of tumors showing DAPI (blue), MHCII+ (red), CD8+ (orange), and Foxp3+ (green) cell infiltration for PBS and β-glucan + *a* PD-L1 groups. Statistical difference was calculated using two-tailed unpaired student’s *t*-test. Data were expressed as means ± *S*. * *p* < 0.05, ** *p* < 0.01 and *** *p* < 0.001.

Encouraged by the results of subcutaneous pancreatic tumor models, the combination therapy approach was employed in a clinically relevant in vivo model to assess the anti-tumor efficiency of β-glucan plus PD-L1 blockade. An orthotopic model of pancreatic cancer was established, these mice were injected with Pan02 cells concatenated with luciferase, which showed the progression of the tumor in vivo was effectively inhibited after combination therapy (Fig. 4A‒B). It is worth mentioning that the combination therapy of β-glucan and anti-PD-L1 may inhibit hepatic metastasis of pancreatic cancer, limited by the sample capacity only shown in one-third mouse (Fig. 4B). Combination therapy also prolonged the survival of Pan02-challenged mice (Fig. 4C). Same as the previous study, in the combination treatment group, much higher expression of serum cytokines IFN-γ plays an important role in cellular immunity against cancer (Fig. 4D). The mIHC displayed that DCs and CTL were significantly increased in the combination therapy group. On the contrary, the Treg showed a lower infiltration in tumors receiving combination therapy (Fig. 4E).

## Discussion

Although microwave ablation is a clinically approved therapeutic technique for advanced pancreatic cancer, tumor recurrence post-microwave ablation remains a question to resolve. To achieve improved microwave ablation therapeutic outcomes, the combinations of ablation with immune checkpoint inhibitors including anti-PD-L1 and anti-CTLA4 against unresectable pancreatic carcinoma have been studied.^25^^,^^26^ However, it has been reported that the inflammation induced after incomplete thermal ablation would promote tumor progression and restrain the therapeutic effect of immunotherapy, via the accumulation of immunosuppressive tumor-associated DCs inside TME.^27^ Therefore, our study develops a more efficient target of immunotherapy to enhance the therapeutic benefit of microwave ablation and thus further extend its clinical value.

A previous study has shown that β-glucan can induce DCs tumoricidal activity and inhibit tumor development.^28^ Compared to traditional immune adjuvants FLT3 Ligand (FLT3L), β-glucan's induction of trained immunity in innate cells provides a stronger anti-tumor potential for combination with PD-L1 blockade. β-glucan epigenetically reprograms DCs for long-term functional hyperresponsiveness, enabling sustained pro-inflammatory pressure within the tumor microenvironment that directly counteracts myeloid-derived immunosuppression. In this research, we found that β-glucan combined with PD-L1 antibody therapy can inhibit pancreatic cancer progression after ablation more strongly than β-glucan alone or PD-L1 antibody alone. Furthermore, we found that β-glucan significantly improved the immunological effect by increasing the concentrations of DCs to modulate the suppressive TME. Recent research has reported that the blockade of interactions between high expression of PD-L1 on DCs and high expression of programmed cell death-1 (PD-1) on T-cells can enhance response and inhibit tolerance of T-cells, playing an important role in tumor immunity.^29^ As our research institute discovered that H3K27Ac in β-glucan-trained DCs drives Th1/CTL responses by promoting cytokine production (e.g., IL-6, TNF-α and IFN-γ), while simultaneously suppressing Treg recruitment. This reverses the “cold” TME phenotype, augmenting T-cell infiltration and sensitivity to PD-L1 inhibition. And trained DCs maintain anti-tumor vigilance, providing continuous innate memory that synergizes with adaptive immunity reinvigorated by PD-L1 blockade.

PD-1 as an immune checkpoint receptor, which expresses on activated T, B and Dendritic (DC), has been used widely in the clinical setting.^30^ Although PD-L1-blocking antibodies has been the common choice in partial tumors, immunotherapy-related resistance limits its application and effect.^31^^,^^32^ To broaden the range of PD-1-related immunotherapy, multiagent cancer therapy combination regimens have been widely researched and explored. In this study, we found that β-glucan is a potent immune adjuvant, can enhance the anti-tumor effect of PD-L1 blocking antibodies via activating DCs maturation.

While our results are promising, several limitations warrant consideration. The long-term durability of the combination's anti-tumor effect, potential for acquired resistance, and long-term safety profile of β-glucan combined with anti-PD-L1, including the risk of hyperinflammation, remain unknown. Furthermore, the limited sample size in the current study impacts the robustness of our conclusions regarding metastasis inhibition. Specifically, the observation of liver metastasis in only one control mouse, while encouraging, necessitates caution due to the small cohort size. A larger sample may reveal the inhibitory ability of combined therapy on the distant metastasis of tumors. Thus, translating this pre-clinical combination to patients faces significant hurdles, including dosing and scheduling, safety and toxicity, and the TME complexity of patients. This study aims to reveal that the treatment of β-glucan combined with anti-PD-L1 can effectively inhibit the in situ recurrence after pancreatic cancer ablation. Future studies will be essential to confirm these promising findings with larger animal numbers and further explore the mechanisms of metastasis inhibition.

Despite these limitations, our findings provide a strong rationale for further investigation that β-glucan could significantly broaden the clinical utility of ICB in conjunction with ablation for pancreatic cancer. In summary, we found that the immune adjuvant β-glucan improved the infiltration of matured DCs, and promoted the increased ratio of CTL/Treg by anti-PD-L1 treated in the TME. Furthermore, combination therapy with β-glucan plus anti-PD-L1 was found to show more effectively inhibit tumor progression after ablation. These results suggest that β-glucan is a potentially useful adjuvant in combination with various immune checkpoint-inhibited therapies in clinical practice.

## Ethics approval and consent to participate

This study was performed in line with the principles of the ARRIVE guidelines. Approval was granted by the Institutional Animal Care and Use Committee of the Laboratory Animal Center of Shanghai Tenth People’s Hospital (No. SHDSYY-2021–2855). Informed consent was obtained from all individual participants included in the study.

## Authors’ contributions

SW and XS took part in all the experiments and in the preparation of the manuscript. HW and JC participated in data collection, assisted with the statistical analysis. SW and BH conceived and designed the study. SW and BH supervised the team, facilitated the acquisition of data, and provided directions for the critical review of the manuscript. All authors contributed to the article and approved the submitted version.

## Funding

The authors declare that no financial support was received for the research, authorship, and/or publication of this article.

## Declaration of competing interest

The authors declare no conflicts of interest.
